# In vivo MRI and ex vivo histological assessment of the cardioprotection induced by ischemic preconditioning, postconditioning and remote conditioning in a closed-chest porcine model of reperfused acute myocardial infarction: importance of microvasculature

**DOI:** 10.1186/s12967-017-1166-z

**Published:** 2017-04-01

**Authors:** Tamás Baranyai, Zoltán Giricz, Zoltán V. Varga, Gábor Koncsos, Dominika Lukovic, András Makkos, Márta Sárközy, Noémi Pávó, András Jakab, Csilla Czimbalmos, Hajnalka Vágó, Zoltán Ruzsa, Levente Tóth, Rita Garamvölgyi, Béla Merkely, Rainer Schulz, Mariann Gyöngyösi, Péter Ferdinandy

**Affiliations:** 1 Department of Pharmacology and Pharmacotherapy, Semmelweis University, Budapest, Hungary; 2 Department of Cardiology, Medical University of Vienna, Vienna, Austria; 3 Department of Biochemistry, University of Szeged, Szeged, Hungary; 4 Department of Biomedical Imaging and Image-guided Therapy, Medical University of Vienna, Vienna, Austria; 5 The Heart and Vascular Center, Semmelweis University, Budapest, Hungary; 6 Institute of Diagnostic Imaging and Radiation Oncology, University of Kaposvár, Kaposvár, Hungary; 7 Department of Radiology, University of Pécs, Pecs, Hungary; 8 Institute of Physiology, Justus Liebig University, Giessen, Germany; 9 Pharmahungary Group, Szeged, Hungary

**Keywords:** Ischemic preconditioning, Ischemic postconditioning, Remote conditioning, Myocardial edema, Area at risk, Ischemia/reperfusion injury

## Abstract

**Background:**

Cardioprotective value of ischemic post- (IPostC), remote (RIC) conditioning in acute myocardial infarction (AMI) is unclear in clinical trials. To evaluate cardioprotection, most translational animal studies and clinical trials utilize necrotic tissue referred to the area at risk (AAR) by magnetic resonance imaging (MRI). However, determination of AAR by MRI‚ may not be accurate, since MRI-indices of microvascular damage, i.e., myocardial edema and microvascular obstruction (MVO), may be affected by cardioprotection independently from myocardial necrosis. Therefore, we assessed the effect of IPostC, RIC conditioning and ischemic preconditioning (IPreC; positive control) on myocardial necrosis, edema and MVO in a clinically relevant, closed-chest pig model of AMI.

**Methods and results:**

Acute myocardial infarction was induced by a 90-min balloon occlusion of the left anterior descending coronary artery (LAD) in domestic juvenile female pigs. IPostC (6 × 30 s ischemia/reperfusion after 90-min occlusion) and RIC (4 × 5 min hind limb ischemia/reperfusion during 90-min LAD occlusion) did not reduce myocardial necrosis as assessed by late gadolinium enhancement 3 days after reperfusion and by ex vivo triphenyltetrazolium chloride staining 3 h after reperfusion, however, the positive control, IPreC (3 × 5 min ischemia/reperfusion before 90-min LAD occlusion) did. IPostC and RIC attenuated myocardial edema as measured by cardiac T2-weighted MRI 3 days after reperfusion, however, AAR measured by Evans blue staining was not different among groups, which confirms that myocardial edema is not a measure of AAR, IPostC and IPreC but not RIC decreased MVO.

**Conclusion:**

We conclude that IPostC and RIC interventions may protect the coronary microvasculature even without reducing myocardial necrosis.

**Electronic supplementary material:**

The online version of this article (doi:10.1186/s12967-017-1166-z) contains supplementary material, which is available to authorized users.

## Background

Despite the wide availability of advanced revascularization techniques, acute myocardial infarction (AMI) is one of the leading causes of mortality and morbidity in developed countries [1]. Preclinical studies of various ischemic conditioning techniques have shown promising results in the reduction of AMI-related cardiac damage (see for an extensive recent review: [2]). Ischemic preconditioning (i.e., brief cycles of ischemia and reperfusion of the involved coronary artery before sustained ischemia; IPreC) was shown first to attenuate subsequent myocardial damage [3]. Since then, it has been demonstrated that ischemic conditioning reduces myocardial ischemia/reperfusion injury (IRI) if applied after cardiac ischemia (ischemic postconditioning; IPostC), which is more feasible for clinical application [4]. Moreover, cardioprotection could be elicited by applying ischemic stimuli on a distant area of the heart or on a remote organ (e.g., kidney, skeletal muscle) termed remote ischemic conditioning (RIC) [5]. Both IPostC and RIC have been reported to be cardioprotective in preclinical [5] and clinical [6–9] settings. However, to date, the largest clinical trials in IPostC did not show any benefit of IPostC on ST-elevation myocardial infarction patients after primary percutaneous intervention (POST and DANAMI 3-iPOST trial) in terms of long-term outcome [10, 11]. So far, a small-scale clinical trial involving ST-elevation myocardial infarction (STEMI) patients has been completed and demonstrated long-term efficacy of RIC as assessed by significant improvement of major adverse cardiac and cerebrovascular events [12]. Nevertheless, two large clinical trials reported that RIC did not improve the long-term outcome after cardiac surgery (ERICCA [13] and RIPHEART [14] trials).

The cardioprotective efficacy of an intervention is assessed by different methods in preclinical settings and in clinical trials. For instance, there is no accurate and widely available method to measure the area at risk (AAR; the area excluded from the coronary circulation), which is the basis of preclinical cardioprotective studies. Although it has been proposed that myocardial edema by T2-weighted magnetic resonance imaging (MRI) correlates well with histopathology-based evaluation of AAR [15], it has been demonstrated that myocardial edema correlates better with the myocardial necrosis than with the AAR [16]. Furthermore, efficacy of interventions in (pre)clinical studies are almost exclusively based on the measurement of myocardial necrosis by assessing infarct size or necroenzyme release, which primarily represents the decay of cardiac myocytes, and which are major determinants of the outcome of AMI according to clinical studies [17]. However, damage to other cell types of the heart, such as the coronary microvasculature may also contribute to the IRI [2, 18]. For instance, myocardial edema [19], microvascular obstruction (MVO; ischemic area, where myocardial perfusion is not restored despite successful revascularization) [20], endothelial dysfunction, and microembolisation are major signs of microvascular damage [21].

Therefore, we studied the effects of IPostC and RIC by comparing them to the effects of the positive control (IPreC) on measures of IRI (myocardial necrosis, edema and MVO) as determined by in vivo MRI and ex vivo histological staining in a clinically relevant, closed-chest swine model of AMI.

## Methods

### Experimental design (Fig. 1) 

Domestic female pigs (25–35 kg; genotype: DanBred hybrid, purchased from the University of Kaposvár, Hungary) were kept according to the Big Dutchman principles. The animals were fed with a pregnant sow diet containing a low energy and balanced protein level produced by Dalmand co. Ltd. They further underwent regular veterinarian check-ups, and only healthy animals were selected for the study. Furthermore, before percutaneous intervention, transthoracic echocardiography, and angiography was served as a baseline screening method to exclude animals with abnormal coronary anatomy or myocardial disease.

**Fig. 1:**
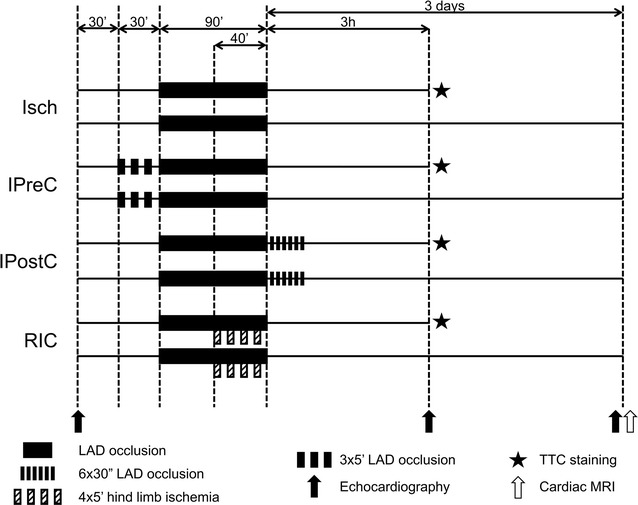
Experimental protocol*. Isch* ischemia only, *IPreC* ischemic preconditioning, *IPostC* ischemic postconditioning, *RIC* remote ischemic conditioning, *LAD* left anterior descendent coronary artery, *TTC* triphenyl tetrazolium chloride, *MRI* magnetic resonance imaging

The pigs were block randomized [22] into four groups: ischemia only (Isch; n = 17), IPreC (n = 12), IPostC (n = 14) and RIC (n = 17). Three animals died during myocardial ischemia (Isch: 1; IPreC: 1; IPostC: 0; RIC: 1) and 4 during reperfusion due to therapy resistant malignant ventricular rhythm disturbances (Isch: 2; IPreC: 1; IPostC: 1; RIC: 0). Additionally, 3 animals were excluded due to procedural technical reasons. The final case numbers were 14, 9, 12 and 14 in Isch, IPreC, IPostC and RIC groups, respectively. According to the current ESC STEMI guidelines [23, 24], the pigs were pretreated with loading doses of 250 mg acetyl salicylic acid and 300 mg clopidogrel with maintenance doses of 100 and 75 mg pro day, respectively. Although 600 mg dose of clopidogrel has been shown to superior over a 300 mg dose in myocardial necrosis reducing effects [25], our experimental protocol has been planned to minimize the effect of possible confounding factors such as clopidogrel [26]. Animals were sedated with 12 mg/kg ketamine hydrochloride, 1 mg/kg xylazine, and 0.04 mg/kg atropine intramuscularly after an overnight fast. Anesthesia was induced by inhalation of isoflurane (2–2.5 vol%). Animals were intubated endotracheally and anesthesia was maintained by inhalation of an isoflurane oxygen mix (2–2.5 vol% and 3 L/min). Magnesium sulphate (4.06 mEq diluted in 10 mL, in every 60 min) and a continuous amiodarone infusion (300 mg diluted in 500 mL saline) were being administered throughout the procedure via an ear vein. Sheaths were inserted into femoral artery and femoral vein to further have entry routes for the catheterization, and 5000–5000 IU heparin was administered via each sheath. Cardiac function was assessed with echocardiography. Baseline hemodynamics were recorded, and selective angiography of the left coronary artery was performed. After the analysis of the baseline angiogram, a balloon catheter (2.75 mm diameter, 8 mm length) (Abbott Vascular) was placed in the mid part of the left anterior descending coronary artery (LAD) after the origin of the 2nd diagonal branch. For induction of AMI, the intracoronary balloon was inflated with 5 atm for 90 min, followed by deflation of the balloon, resulting in reperfusion (3 h or 3 days) which was confirmed by coronarography. In IPreC group, LAD was occluded by the inflation of the balloon at 5 atm 3 times for 5 min followed by 5 min of reperfusion, while in other animals the balloon was left deflated for 30 min [27]. Then LAD was occluded by inflating coronary balloon, which was confirmed by coronarography. RIC was performed by 4 cycles of 5 min occlusion and 5 min reperfusion of the femoral vessels by tightening and releasing of a snare around the right hind limb starting at the 50th min of LAD occlusion [9]. We verified the hind limb ischemia in three ways: (1) Apparent lividity during ischemia and pronounced hyperemia during reperfusion was observed distal to the occlusion. (2) In each animal, a superficial femoral artery was cannulated distal to the occlusion, and blood pressure was measured during the intervention. The minimum of 30/30 mmHg blood pressure was achieved while the wire was tightened around the hind limb. (3) In one particular animal, we performed a femoral angiography before and during hind limb ischemia as well [Additional file 1: Video_S1.avi (before), Additional file 2: Video_S2.avi (during hind limb ischemia)]. IPostC was initiated within 1 min after the termination of 90-min index ischemia and was performed by applying 6 cycles of 30/30 s LAD occlusion and reperfusion [28]. The interventional cardiologist was not blinded during the investigation due to the nature of the procedure: IPreC and IPostC were achieved by inflating a balloon in the LAD, which required an unblinded interventional cardiologist. Nonetheless, the interventional cardiologist was not aware of the allocation until the initiation of the experimental intervention (e.g. right after balloon deflation if the animal was in IPostC group). After reperfusion was initiated 5000 IU heparin was given intracoronary. Final reperfusion was confirmed with coronarography and the catheters were removed. Ten min after the initiation of reperfusion, the hemodynamic data was recorded again. Anesthesia was either maintained for 3 h or in case of 3 days reperfusion, wounds were closed and anesthesia was terminated by the withdrawal of isoflurane. Analgesia was applied by intramuscular injections of 1 g metamizole. An antibiotic cocktail (100 mg benzathine benzylpenicillin, 100 mg procaine benzylpenicillin, 200 mg dihydrostreptomycin-sulphate) was given i.m. before recovery.

### Measurement of myocardial necrosis, edema and MVO by cardiac MRI

The amount of myocardial necrosis and MVO were determined by acquiring late gadolinium-enhanced images (gold standard method) [9, 29]. Dark blood prepared IR-TSE sequence single slice breath-hold acquisition T2w protocol was used to detect myocardial edema [9, 29]. 3 days (70–78 h) after the deflation of the balloon in LAD, anesthesia was induced by inhalation of an isoflurane-oxygen mix. Prior to the cardiac MRI, atracurium was administered and ventilation was maintained with mechanical ventilation. For the assessment of myocardial necrosis and edema, cardiac MRI was performed using a 1.5T clinical scanner (Avanto, Siemens) using a phased array coil and a vector ECG system. Cine MRI images was acquired using a retrospectively ECG-gated, steady-state free precession cine MRI technique (Cinetruefisp sequence) in short-axis and long-axis views of the heart using 1.2 ms echo time, 40 ms repetition time, 65° flip angle, 15 segments, 360 mm field-of-view, 8 mm slice thickness, and 256 × 256 image matrix. The image resolution was 1.4 × 1.4 × 8 mm. T2-weighted cardiac MRI was performed by short inversion time inversion recovery dark blood technique with single slice breath-hold acquisition and inversion recovery preparation (TI = 170 ms; 15 segments; every second trigger pulse; TE 74 ms, flip angle 180°). The slice position and resolution was identical as cine images. The late gadolinium-enhanced images were acquired to determine the amount of myocardial necrosis and MVO. A 2-dimensional single shot Truefisp sequence with non-selective IR pulse shift acquisition to a diastolic phase of the cardiac cycle by adjusting the TR was used 12–15 min after administration of a gadolinium-based contrast agent (0.13 mmol/kg gadobutrol, Gadovist 1.0 mmol, Bayer), with slice positions identical to the cine images. Typical in-plane resolution was 1.4 × 1.4 × 8 mm (echo time 1.2 ms, flip angle 50°, triggering to every other heart beat). The inversion time was set to null the signal of viable myocardium and ranged from 280–320 ms. Left and right ventricular end-diastolic and end-systolic volumes, stroke volumes, ejection fractions, and masses were quantified using manual planimetry of end-diastolic and end-systolic short-axis SSFP cine images with MASS 7.6 analysis software (Magnetic Resonance Analytical Software System, Medis Medical Imaging Software, Leiden, The Netherlands). Myocardial necrosis and edema were quantified after manual planimetry both on the delayed contrast enhancement and T2-weighted images by delineation of myocardium with signal intensity 4 SDs above the mean signal obtained in the remote noninfarcted myocardium using MASS 7.6 analysis software. If present, the hypointense zone in the center of the hyperenhancement (MVO) was quantified and added to the infarct volume as previously described [30]. Values were expressed relative to the left ventricular mass. The measurement of MVO with late gadolinium enhancement closely correlates with myocardial contrast echocardiography, angiographic and invasive indices used for the assessment of MVO [31]. Moreover, late-gadolinium enhancement correlates with the histological measurement of MVO as demonstrated by Zalewski et al. [29].

### Myocardial necrosis and AAR measurement by ex vivo staining

Hearts were removed from the chest, and placed immediately in ice-cold saline 3 h after reperfusion. LAD was reoccluded, ex vivo at the same place as in vivo (prior to the obduction, coronary angiography of the pig was reviewed, and the occlusion site was identified.), and Evans blue (Sigma) was injected into the coronary arteries through their orifices to negatively stain the AAR [29, 32]. 10 mm slices were then cut and incubated in 1% triphenyltetrazolium chloride (TTC, Sigma) at 37 °C for 15 min to stain viable areas. After overnight fixation with 4% formalin, slices were weighed and scanned for blind planimetric analysis (InfarctSize 2.4b software; Pharmahungary Group). AAR was expressed relative to the left ventricular (LV) mass, and myocardial necrosis relative to the AAR mass.

### Coronary angiography and AAR calculation

All animals underwent coronary angiography according to the protocol established by the catheterization laboratory. Anterograde flow in the artery before and after balloon inflation was characterized using the TIMI (Thrombolysis in Myocardial Infarction) system [33]. TIMI myocardial perfusion grade and myocardial blush grade were assessed visually on the angiogram and made by expert interventional cardiologist, and all data were entered prospectively into a database. Myocardial blush grade has been defined as follows: 0, no myocardial blush or contrast density; 1, minimal myocardial blush or contrast density; 2, moderate myocardial blush or contrast density but less than that obtained during angiography of a contralateral or ipsilateral non–infarct-related coronary artery; and 3, normal myocardial blush or contrast density, comparable with that obtained during angiography of a contralateral or ipsilateral non–infarct-related coronary artery. When myocardial blush persisted (“staining”), this phenomenon suggested leakage of contrast medium into the extravascular space and was graded 0 [34, 35]. No digital techniques were used. The AAR was established by using the modified APPROACH score [36].

### Transthoracic echocardiography

Two-dimensional, M-mode and Doppler echocardiographic examinations were performed in accordance with the criteria of the American Society of Echocardiography with a Vivid i portable ultrasound system (General Electric Medical Systems) using a phased array 2.7–8 MHz transducer (6S-RS probe). Data of three consecutive heart cycles were analysed (EchoPac Dimension software; General Electric Medical Systems) in a blinded manner. The mean values of three measurements were calculated and used for statistical evaluation.

### Statistics

Values were expressed as *mean* ± *standard error of mean*. Statistical analyses were done by using *one way* or *repeated measures ANOVA* with *LSD* or *Dunnett’s* post hoc *test* and *Kruskal*–*Wallis test* as indicated (IBM SPSS Statistics, Version 19). To limit the case numbers in the cardiac MRI study, *one*-*way ANOVA* was performed by using bootstrapping with 1000-sample replacement [37], as frequently used in clinical studies [38–40] and recommended for preclinical studies as well [41]. Statistical significance was accepted if the p value was below 0.05.

## Results

### Myocardial necrosis, edema and MVO by cardiac MRI

**Fig. 2:**
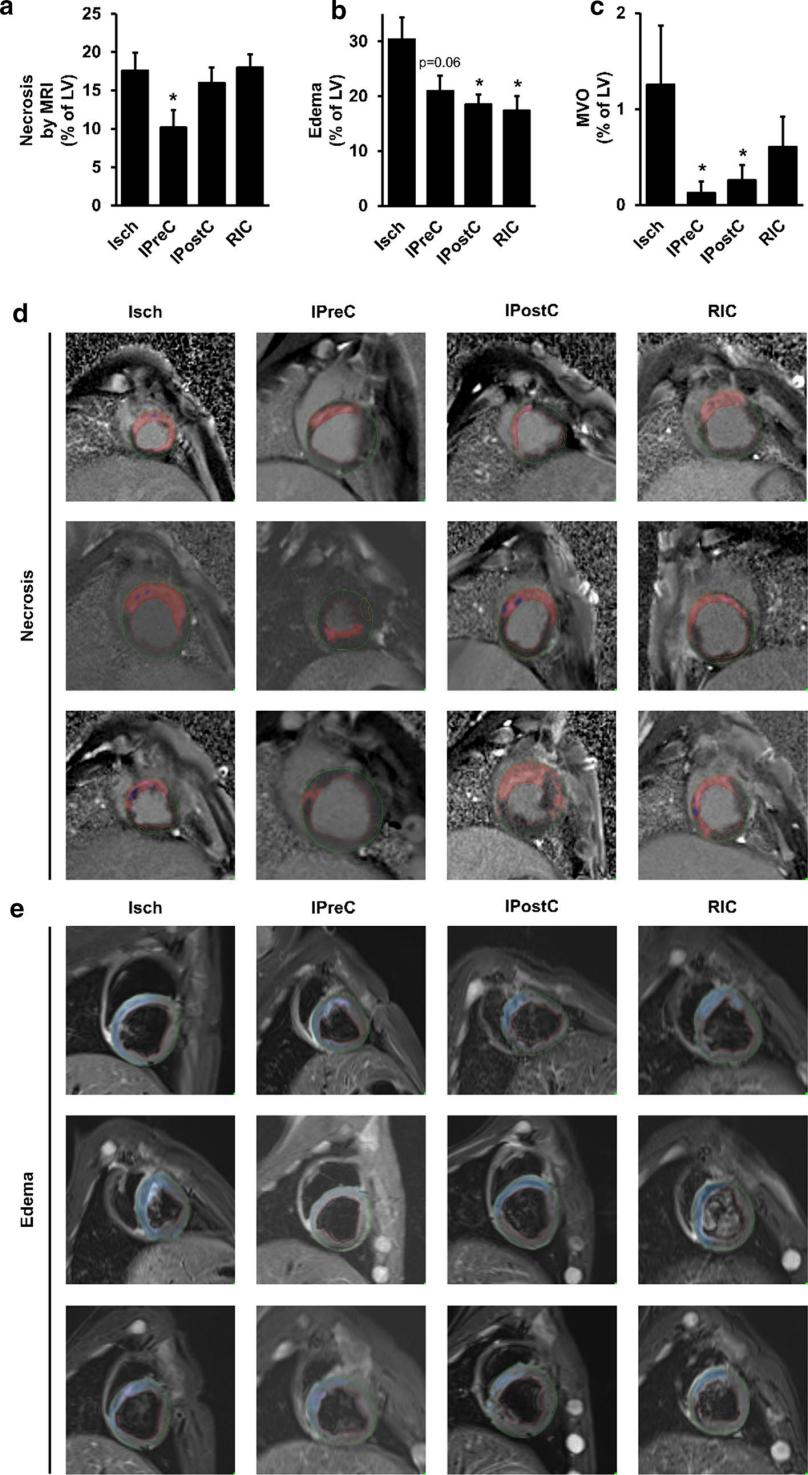
The effect of IPreC, IPostC and RIC on myocardial necrosis (**a**; n = 4–8/group), edema (**b**; n = 4–9/group) and MVO (**c**; n = 4–8/group) size as evaluated with cardiac MRI. **d-e** Representative MRI images of myocardial necrosis and edema. *Green line*: apicardial outline, *red line*: endocardial outline, *red area*: myocardial necrosis, *dark blue area*: MVO, *light blue area*: myocardial edema. *p < 0.05 vs. Isch. *Isch* ischemia only, *IPreC* ischemic preconditioning, *IPostC* ischemic postconditioning, *RIC* remote ischemic conditioning, *MRI* magnetic resonance imaging, *LV* left ventricle, *MVO* microvascular obrstruction

To quantify myocardial necrosis, edema and MVO, cardiac MRI was performed 3 days after coronary occlusion and reperfusion. Myocardial necrosis (% of LV) was not affected by IPostC and RIC (n = 7, and 8, respectively), however, IPreC (n = 4) attenuated it significantly as compared to the Isch group (n = 7), although only 4 IPreC cases were studied with MRI (Fig. 2a). Myocardial edema (% of LV) was significantly decreased by IPostC and RIC (n = 9, and 7, respectively) as compared to the Isch group (n = 7), however, only a tendency of decrease was observed by IPreC (n = 4; p = 0.06; Fig. 2b). At baseline, hemodynamic parameters were not significantly different between groups, whereas at reperfusion heart rate was significantly lower in IPreC and RIC groups as compared to the Isch group (Table 1). AAR based on angiographic score was not different between groups (Table 2). Furthermore, angiographic measures of reperfusion, i.e., TIMI score, myocardial blush grade, and TIMI myocardial perfusion grade were not affected by conditioning stimuli (Table 2). MVO volume (% of LV) was significantly decreased by IPreC and IPostC (n = 4, and 7, respectively), but not by RIC (n = 8) as compared to the Isch group (n = 7) (Fig. 2c).

**Table 1:** Hemodynamic data

	Baseline					Reperfusion 10 min				
	HR (1/min)	SBP (mmHg)	DBP (mmHg)	MABP (mmHg)	dP/dt (mmHg/s)	HR (1/min)	SBP (mmHg)	DBP (mmHg)	MABP (mmHg)	dP/dt (mmHg/s)
Isch	109.5 ± 5.2	112.6 ± 5.9	69.3 ± 4.6	87.4 ± 5.1	1037 ± 81	130.2 ± 5.1	93.6 ± 6.4	54.1 ± 4.0	71.1 ± 4.6	800 ± 54
IPreC	90.6 ± 9.4	102.0 ± 10.8	65.5 ± 8.1	82.6 ± 7.2	838 ± 141	92.4 ± 14.4*	82.2 ± 4.4	53.8 ± 4.7	63.4 ± 3.8	560 ± 196
IPostC	103.7 ± 5.2	117.7 ± 7.3	74.4 ± 5.1	92.2 ± 6.0	968 ± 110	116.8 ± 6.1	93.3 ± 5.0	50.4 ± 2.2	68.1 ± 3.0	764 ± 87
RIC	106.2 ± 6.6	107.5 ± 6.5	67.8 ± 3.8	85.2 ± 5.1	976 ± 91	113.9 ± 4.4*	86.5 ± 4.6	50.0 ± 2.2	65.2 ± 3.0	647 ± 53

* p < 0.05 vs. Isch. n = 4–13/group

*Isch* ischemia only, *IPreC* ischemic preconditioning, *IPostC* ischemic postconditioning, *RIC* remote ischemic conditioning, *HR* heart rate, *SBP* systolic blood pressure, *DBP* diastolic blood pressure, *MABP* mean arterial blood pressure

**Table 2:** Angiographic evaluation

	APPROACH score (% of LV)	TIMI score	Myocardial blush grade	TIMI myocardial perfusion grade
Isch	19.2 ± 2.2	2.4 ± 0.3	2.5 ± 0.3	2.3 ± 0.3
IPreC	18.6 ± 2.4	2.2 ± 0.3	2.4 ± 0.3	2.3 ± 0.3
IPostC	24.2 ± 2.2	1.8 ± 0.3	2.0 ± 0.4	2.0 ± 0.4
RIC	19.7 ± 2.5	2.3 ± 0.3	2.5 ± 0.3	2.5 ± 0.3

n = 8–12/group

*Isch* ischemia only, *IPreC* ischemic preconditioning, *IPostC* ischemic postconditioning, *RIC* remote ischemic conditioning, *LV* left ventricle

### Myocardial necrosis and AAR evaluated by ex vivo staining

**Fig. 3:**
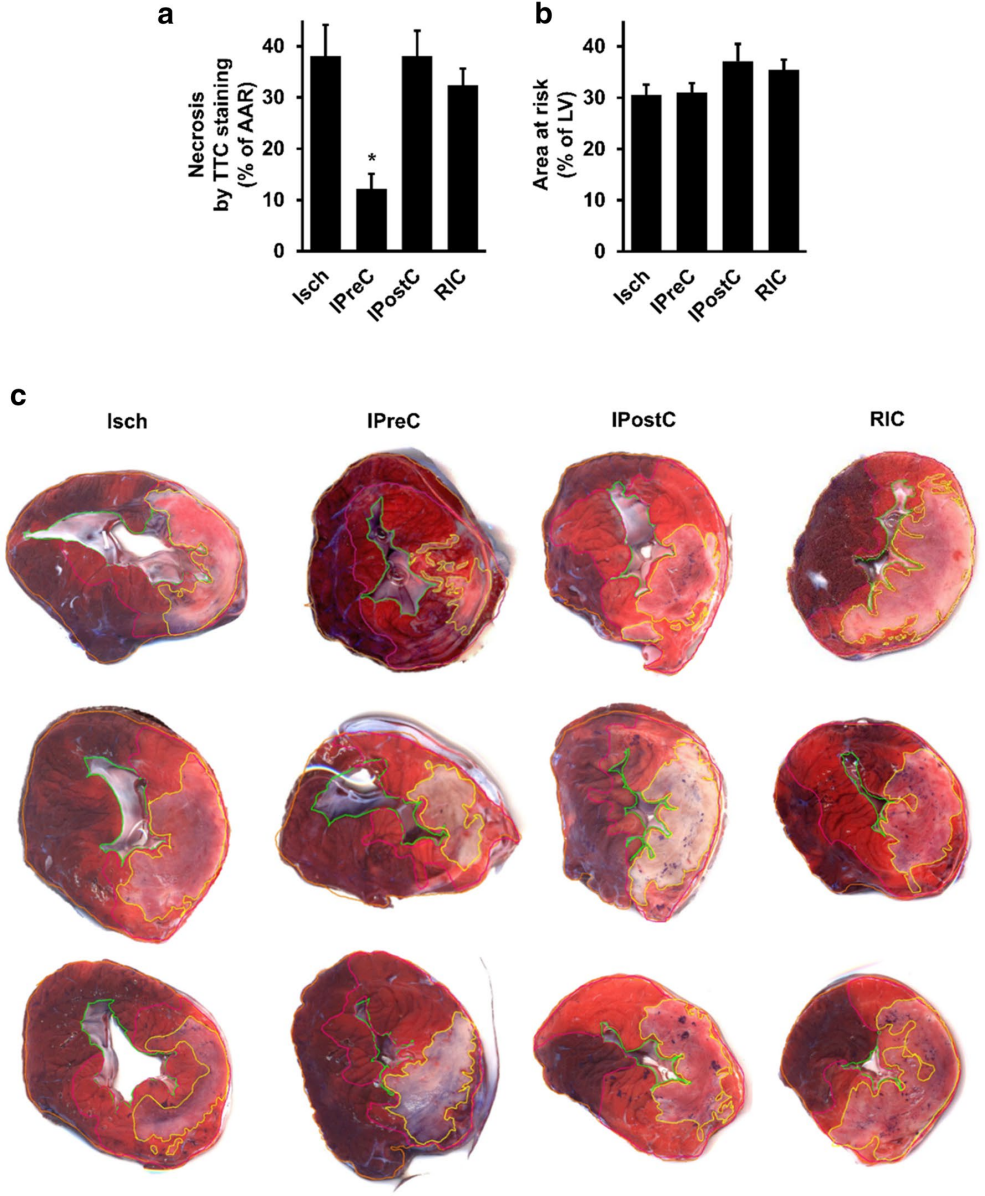
The effect of IPreC, IPostC and RIC on myocardial necrosis (**a**; n = 5–6/group) and AAR (**b**; n = 5–6/group) as evaluated with conventional TTC and *Evans blue* staining. **c** Representative images of* Evans blue*/TTC-stained heart sections from three different hearts indicating myocardial necrosis and AAR (Each image was taken from the apical side of the third 1-cm slice. Images are optimized for visualization of TTC staining in the InfarctSize software.). *Orange* slice outline, *green* ventricular chamber, *purple* AAR, *yellow* myocardial necrosis. *p < 0.05 vs. Isch. *Isch* ischemia only, *IPreC* ischemic preconditioning, *IPostC* ischemic postconditioning, *RIC* remote ischemic conditioning, *AAR* area at risk, *TTC* triphenyl tetrazolium chloride, *MRI LV* left ventricle

To assess myocardial necrosis and AAR by an ex vivo histological method, the gold standard TTC and Evans blue staining were applied after 3 h of reperfusion. IPostC and RIC did not decrease myocardial necrosis (n = 5, and 5, respectively) (% of AAR), however, IPreC (n = 6), significantly decreased it as compared to the Isch group (n = 5) (Fig. 3a). There was no difference in AARs between the experimental groups (% of LV) as evaluated by Evans blue staining (Fig. 3b).

### Myocardial function by cardiac MRI and echocardiography

Myocardial function was analyzed by cardiac MRI and echocardiography. Myocardial function was not different between groups after either 3 h or 3 days of reperfusion (Tables 3, 4, 5).

**Table 3:** Myocardial function as assessed by MRI

	Isch	IPreC	IPostC	RIC
HR (1/min)	96.26 ± 7.76	100.50 ± 5.06	103.11 ± 4.75	100.13 ± 5.07
LV EDVI (mL/kg)	3.63 ± 0.25	3.10 ± 0.02	3.55 ± 0.16	3.55 ± 0.09
LV ESVI (mL/kg)	2.07 ± 0.15	1.81 ± 0.14	1.99 ± 0.13	2.09 ± 0.17
LV SVI (mL/kg)	1.49 ± 0.11	1.29 ± 0.15	1.53 ± 0.07	1.40 ± 0.10
LV EF (%)	43.47 ± 1.67	41.64 ± 4.75	44.63 ± 1.55	41.55 ± 2.77
LV COI (L/min/kg)	0.15 ± 0.02	0.11 ± 0.01	0.16 ± 0.01	0.14 ± 0.02
RV EDVI (mL/kg)	2.86 ± 0.21	2.80 ± 0.18	2.43 ± 0.09	2.59 ± 0.10
RV ESVI (mL/kg)	1.40 ± 0.09	1.65 ± 0.05	1.24 ± 0.07	1.32 ± 0.08
RV SVI (mL/kg)	1.46 ± 0.15	1.15 ± 0.18	1.29 ± 0.07	1.28 ± 0.08
RV EF (%)	43.95 ± 2.43	40.63 ± 3.66	47.44 ± 2.89	47.42 ± 2.61
RV COI (L/min/kg)	0.14 ± 0.02	0.09 ± 0.01	0.13 ± 0.01	0.12 ± 0.01

n = 4–8/group

*Isch* ischemia only, *IPreC* ischemic preconditioning, *IPostC* ischemic postconditioning, *RIC* remote ischemic conditioning, *LV* left ventricle, *RV* right ventricle, *EDVI* end-diastolic volume index, *ESVI* end-systolic volume index, *SVI* stroke volume index, *EF* ejection fraction, *COI* cardiac output index

**Table 4:** Cardiac function parameters measured by echocardiography before interventions and after 3 h of reperfusion

	Isch		IPreC		IPostC		RIC	
	Baseline	3 h	Baseline	3 h	Baseline	3 h	Baseline	3 h
LV EDDI (mm/kg)	1.46 ± 0.10	1.54 ± 0.06	1.54 ± 0.10	1.54 ± 0.08	1.46 ± 0.08	1.46 ± 0.11	1.32 ± 0.10	1.44 ± 0.16
LV ESDI (mm/kg)	1.02 ± 0.11	1.21 ± 0.06	1.17 ± 0.10	1.21 ± 0.07	1.06 ± 0.07	1.09 ± 0.14	0.89 ± 0.11	1.09 ± 0.18
LV FS (%)	31.48 ± 3.87	23.83 ± 3.85	24.50 ± 2.91	21.85 ± 1.59	27.88 ± 1.80	27.13 ± 6.28	33.47 ± 4.47	28.04 ± 3.69
LV EF (%)	58.78 ± 6.04	37.90 ± 3.66*	47.60 ± 4.58	44.00 ± 2.67	53.75 ± 2.93	48.29 ± 8.29	60.07 ± 5.89	51.13 ± 6.76
LV EDVI (mL/kg)	3.07 ± 0.34	3.37 ± 0.26	3.37 ± 0.43	3.52 ± 0.49	3.14 ± 0.31	3.21 ± 0.45	2.53 ± 0.42	3.26 ± 0.78
LV ESVI (mL/kg)	1.41 ± 0.31	1.67 ± 0.25	1.61 ± 0.28	2.14 ± 0.32	1.47 ± 0.19	1.80 ± 0.40	1.09 ± 0.33	2.10 ± 0.81
LV SVI (mL/kg)	1.66 ± 0.14	1.41 ± 0.09	1.76 ± 0.32	1.38 ± 0.23	1.67 ± 0.18	1.41 ± 0.20	1.44 ± 0.18	1.31 ± 0.16
LV DMI (g/kg)	4.90 ± 0.61	4.87 ± 0.52	5.78 ± 0.99	4.73 ± 0.73	4.11 ± 0.60	4.52 ± 0.36	4.80 ± 0.55	4.53 ± 0.50
LV SMI (g/kg)	4.91 ± 0.63	5.03 ± 0.75	6.63 ± 1.29	5.10 ± 0.97	4.51 ± 0.82	4.34 ± 0.50	4.56 ± 0.75	4.99 ± 0.83

* p < 0.05 vs. corresponding baseline. n = 9–10/group

*LV* left ventricle, *EDDI* end-diastolic diameter index, *ESDI* end-systolic diameter index, *FS* fractional shortening, *EF* ejection fraction, *EDVI* end-diastolic volume index, *ESVI* end-systolic volume index, *SVI* stroke volume index, *DMI* diastolic mass index, *SMI* systolic mass index

**Table 5:** Cardiac function parameters measured by echocardiography before interventions and after 3 days of reperfusion

	Isch		IPostC		RIC	
	Baseline	3 days	Baseline	3 days	Baseline	3 days
LV EDDI (mm/kg)	1.50 ± 0.09	1.47 ± 0.14	1.55 ± 0.08	1.59 ± 0.10	1.44 ± 0.05	1.47 ± 0.03
LV ESDI (mm/kg)	1.07 ± 0.11	1.05 ± 0.11	1.12 ± 0.09	1.08 ± 0.07	1.00 ± 0.04	0.99 ± 0.04
LV FS (%)	29.07 ± 3.92	28.50 ± 2.70	28.10 ± 3.16	31.38 ± 2.26	30.33 ± 2.05	32.93 ± 2.21
LV EF (%)	54.93 ± 5.53	54.67 ± 3.87	54.14 ± 4.49	59.48 ± 2.76	57.67 ± 2.99	61.04 ± 3.07
LV EDVI (mL/kg)	3.04 ± 0.30	3.29 ± 0.31	3.24 ± 0.29	3.59 ± 0.44	2.74 ± 0.16	3.05 ± 0.11
LV ESVI (mL/kg)	1.44 ± 0.28	1.48 ± 0.16	1.56 ± 0.24	1.41 ± 0.18	1.25 ± 0.15	1.19 ± 0.10
LV SVI (mL/kg)	1.59 ± 0.11	1.62 ± 0.29	1.68 ± 0.14	2.18 ± 0.31	1.66 ± 0.14	1.86 ± 0.12
LV DMI (g/kg)	4.43 ± 0.29	3.53 ± 0.45	3.99 ± 0.43	4.12 ± 0.44	4.16 ± 0.31	4.03 ± 0.26
LV SMI (g/kg)	4.31 ± 0.25	3.94 ± 0.35	3.96 ± 0.36	4.36 ± 0.44	3.66 ± 0.30	4.19 ± 0.23

n = 7–9/group

*LV* left ventricle, *EDDI* end-diastolic diameter index, *ESDI* end-systolic diameter index, *FS* fractional shortening, *EF* ejection fraction, *EDVI* end-diastolic volume index, *ESVI* end-systolic volume index, *SVI* stroke volume index, *DMI* diastolic mass index, *SMI* systolic mass index

## Discussion

Here we studied the effect of IPostC and RIC on IRI-related MRI parameters in a clinically relevant, closed-chest porcine model of reperfused AMI and demonstrated that IPostC and RIC protected the microvasculature against IRI, as they reduced myocardial edema, and IPostC decreased MVO. However, IPostC or RIC did not reduce myocardial necrosis, although the positive control IPreC reduced it. This is the first comparative demonstration of the variable effects of different conditioning maneuvres on IRI-related MRI parameters, and that myocardial edema and MVO change independently from myocardial necrosis. Our results support findings of clinical trials that myocardial edema may change due to various interventions and it does not represent AAR.

The translation of cardioprotective conditioning stimuli into the clinical practice has been proven difficult and disappointing despite numerous positive proof-of-concept clinical trials [18]. The neutral results have been attributed to many factors, such as recruitment of inadequate patient population, type of revascularization, inclusion of late revascularizations, comorbidities and comedications [18]. Moreover, the strict adherence to certain endpoints, such as myocardial infarct size and the neglect of the microvasculature might also hinder the successful translation of various cardioprotective strategies [21]. Here we demonstrated with ex vivo Evans blue staining and in vivo angiography scoring that AAR was not affected by conditioning stimuli, while myocardial edema was significantly decreased by both IPostC and RIC indicating that the extensive damage of the cardiac microvasculature was prevented. Similarly, two clinical trials showed that myocardial edema is attenuated by IPostC [6] or RIC [9], however, others reported otherwise (IPostC: [42, 43]; RIC: [8, 43]). These results indicate that edema might be independent from AAR in cardioprotection studies, therefore, applying myocardial edema as AAR may lead to false conclusions in clinical trials. To date, no clinical trial has been conducted to reveal the prognostic role of myocardial edema in ST-segment elevation AMI, although it has been shown that non-STEMI patients with myocardial edema had higher mortality [44]. However, it is well-established that the volume or even the presence of MVO, another clinically detectable marker of microvascular injury, correlates with long-term outcome of AMI (see for review [20]). In our present experimental model, IPostC, but not RIC, reduced MVO. Similarly to our findings, it has been reported in a clinical trial that IPostC reduces MVO [45], however, no other clinical trials confirmed the MVO-reducing ability of either IPostC [42, 43] or RIC [9, 43]. Nevertheless, the assessment of myocardial edema and MVO in preclinical and clinical studies may provide additional valuable indices. In our present study, we observed that myocardial function was not different between groups after reperfusion, although IPreC reduced myocardial necrosis. Our results are in line with a number of ischemic conditioning studies in translational models or in clinical trials. IPostC/RIC has been shown to reduce myocardial necrosis after AMI in porcine models, although myocardial function was not different between groups [29, 46, 47]. Similarly, White et al. demonstrated in a clinical trial that RIC did not improve early cardiac function after STEMI, while myocardial necrosis was significantly attenuated by RIC [9]. Furthermore, it seems that early post-AMI cardiac function is not necessarily a good predictor for the future outcome in ischemic conditioning translational studies: For instance, Munk et al. showed that RIC does not influence cardiac function at day 1 after AMI, however, cardiac function was improved at day 30 [48]. In summary, acute post-AMI myocardial function may be determined by many other facts than myocardial necrosis, such as, e.g., actual sympathetic tone, regional wall motion [49], collateral flow [50], transmurality of infarction [50].

Translational models of AMI have a major importance to develop interventions for the clinical practice [18, 51, 52]. For this purpose, pigs are excellent model animals, since their cardiac anatomy and cardiovascular physiology exhibit similarities to the human heart [53]. Although the pig is suitable for closed-chest experimentation, the majority of the studies on the effect of ischemic conditioning on AMI was performed by using open-chest models [32, 54], and relatively few studies are available in closed-chest models. Previously, IPostC [55, 56] and RIC [57, 58] have been shown to reduce myocardial necrosis in closed-chest swine models of AMI. However, in our present experimental conditions, IPostC and RIC did not reduce myocardial necrosis, but the positive control IPreC did. The discrepancy between our results and those of others might be explained by the significantly different perioperative medication and experimental design. The abovementioned studies applied medications required only for the perioperative procedures (e.g. anesthesia, pain control), however, the therapeutical management of AMI consists of other drugs as well. Here we treated animals with acetylsalicylic acid and clopidogrel according to the clinical guidelines [59, 60]. However, it has been shown that COX-2 is an essential mediator of IPreC [61] and of IPostC [62], and that its blockade neutralizes the cardioprotective effect of late IPreC [63]. Similarly, clopidogrel, a P2Y_12_ antagonist, has been retrospectively shown to reduce cardiac necrosis and to decrease cardiovascular events after AMI in clinical trials [64, 65], which might be attributed to its antiplatelet activity and a direct cardioprotective effect. Yang et al. demonstrated that IPostC did not further reduce infarct size when the P2Y_12_ antagonist, cangrelor-pretreatment was applied in rabbits [26]. However, cardioprotection could be elicited by an extended, 8-cycle-long IPostC in closed-chest pigs pretreated with acetylsalicylic acid and clopidogrel [29]. These data indicate that to ensure translational value of animal studies it is essential to apply perioperative medication according to clinical guidelines [66].

In our present study, anesthesia was maintained by isoflurane. It is well-documented that certain anaesthetics, such as fluranes, induce cardioprotection [67] and/or interfere with cardioprotective interventions [68] making the assessment of the effect of conditioning stimuli more difficult. In our present study, we observed a decrease in myocardial necrosis by IPreC but not by IPostC and RIC with isoflurane anesthesia. Although studies showed cardioprotective efficacy of remote ischemic postconditioning with the use of isoflurane [58], and of IPostC after a low-flow index ischemia with enflurane anesthesia in closed-chest porcine models [54], our results show no cardioprotection by IPostC and RIC. This discrepancy might be explained by significant differences between experimental protocols. In conclusion, there is a huge body of evidences that the application of antiplatelet drugs and inhalative anesthetics may interfere with various conditioning maneuvres, although we did not specifically investigate them in our present study.

Although here we assessed the cardioprotective effect of IPostC and RIC as well as the positive control IPreC in a clinically relevant closed-chest pig model of AMI, this study has some limitations. It has been recently shown that the extent of myocardial edema has two peaks over time in swine [69], but not in humans [70]. Therefore, in this study alteration in myocardial edema at day 3 might not necessarily reflect attenuation of edema by conditioning, but the altered dynamics of edema. A long-term follow up would provide data more relevant to the design of clinical studies. Therefore, here we could only speculate whether attenuation of myocardial edema by IPostC and RIC could be interpreted as a valid marker of the measure of IRI, or whether improved long-term outcome depends only on the reduction of myocardial necrosis. Although we did not measure myocardial edema directly (e.g., freeze-dry method), it is generally accepted that T2-hyperintensity correlates well with the myocardial water content, and T2-hyperintensity is excellent for tracking the differences in myocardial edema [19]. Furthermore, according to the clinical routine, cardiac MRI was performed at day 3 [9], whereas ex vivo histopathological staining at 3 h of reperfusion according to the preclinical standards [28]. Therefore, myocardial edema and definite AAR, i.e., Evans blue staining, were evaluated in separate groups. However, we calculated APPROACH score to estimate AAR in the same cohort, in which cardiac MRI was performed. Although APPROACH score system has not been validated in pigs, it is most plausible that it is suitable for the estimation of AAR in pig AMI experiments as well, since several anatomical studies demonstrated that the coronary anatomy of the pig shares a high similarity with that of the human heart (e.g., anastomoses, blood supply territory etc.) [53, 71].

## Conclusions

We compared the cardioprotective efficacy of IPostC and RIC and the positive control IPreC in a clinically relevant, closed-chest porcine model of reperfused AMI for the first time in the literature by using in vivo MRI imaging and ex vivo histology methods. IPostC and RIC did not decrease myocardial necrosis in our model, however, the positive control IPreC reduced it. The coronary microvascular system has been protected by both IPostC and RIC as they attenuated myocardial edema, and IPostC reduced MVO. Our results indicate that parameters of microvascular protection may be important to assess IRI and these parameters might change independently from that of myocardial necrosis. Furthermore, since the intact microcirculation projects improved long-term outcome, its careful evaluation might help to avoid false negative results in preclinical or clinical studies of cardioprotection.

## Additional files

**Additional file 1.** Angiographic imaging of the porcine inguinal vasculature, when the wire was not tightened.

**Additional file 2.** Angiographic imaging of the porcine inguinal vasculature, when the wire was tightened.

## Abbreviations

AAR: area at risk
AMI: acute myocardial infarction
IPostC: ischemic postconditioning
IPreC: ischemic preconditioning
IRI: ischemia/reperfusion injury
LAD: left descending coronary artery
LV: left ventricle
MRI: magnetic resonance imaging
MVO: microvascular obstruction
RIC: remote ischemic conditioning
STEMI: ST-elevation myocardial infarction
TTC: triphenyltetrazolium chloride
